# *c-Myc* Links TGF-β Signaling to Metabolic Control of HIV Reactivation in OM10.1 Cells

**DOI:** 10.3390/microorganisms14102368

**Published:** 2026-10-16

**Authors:** Jakob Harrison-Gleason, Jinhee Kim, Elena Martinelli

**Affiliations:** Department of Medicine, Division of Infectious Diseases, Feinberg School of Medicine, Northwestern University, Chicago, IL 60611, USA

**Keywords:** HIV, SIV, TGF-β, Myc, immunometabolism

## Abstract

Antiretroviral therapy (ART) suppresses HIV replication but does not eliminate persistent viral reservoirs, which can rapidly reseed viremia following treatment interruption. We previously showed that blockade of transforming growth factor beta (TGF-β) signaling in vivo promotes SIV latency reversal and is associated with reversal of cellular metabolic quiescence. Here, using the provirally infected promyelocytic OM10.1 cell line, we investigated the cell-intrinsic mechanisms through which TGF-β regulates HIV reactivation. TGF-β inhibited proviral reactivation following stimulation with distinct latency-reversing agents, and this effect was reversed by pharmacologic inhibition of TGF-β signaling. TGF-β reduced HIV transcription without suppressing TNF-α-induced NF-κB activation, suggesting inhibition through an alternative mechanism. Bulk RNA sequencing revealed broad suppression of *c-Myc*-associated pathways, oxidative phosphorylation, and mTORC1 signaling. Functional metabolic profiling confirmed marked reductions in both glycolytic and mitochondrial ATP production and decreased mitochondrial membrane polarization. Consistent with a functional link between metabolism and viral reactivation, mitochondrial uncoupling strongly inhibited HIV expression. Finally, MYC overexpression completely rescued HIV transcription and partially restored p24 production in TGF-β-treated cells. Together, these findings identify a cell-intrinsic mechanism through which TGF-β restrains HIV reactivation by imposing a metabolically quiescent state, mediated at least in part through suppression of *c-Myc*.

## 1. Introduction

Modern antiretroviral therapy (ART) can durably suppress human immunodeficiency virus type 1 (HIV-1) replication but does not eliminate persistent viral reservoirs, from which viremia can rapidly rebound following treatment interruption. These reservoirs necessitate lifelong therapy and remain the major barrier to an HIV cure [1]. Their persistence is governed by multiple, overlapping mechanisms, including transcriptional and epigenetic regulation, cellular differentiation and activation state, cytokine signaling, and the tissue microenvironment [2]. Growing evidence implicates transforming growth factor beta (TGF-β) as one regulator of these processes [3].

TGF-β is a pleiotropic immunoregulatory cytokine with potent effects on cellular activation, differentiation, and quiescence. In CD4^+^ T-cells, TGF-β promotes regulatory T-cell differentiation through induction of FOXP3 [4], and directly increases the threshold for T cell activation, thereby contributing to maintenance of a quiescent state [5]. TGF-β also strongly shapes myeloid-cell differentiation and function [6], including tissue-resident macrophage programs [7], and is a central regulator of immune homeostasis at mucosal surfaces [8]. These effects are particularly relevant to HIV persistence, which occurs within diverse cellular and tissue environments in which the activation and metabolic state of infected cells can strongly influence viral transcription [9].

Several lines of evidence directly link TGF-β signaling to HIV/SIV persistence. TGF-β is a common component of in vitro primary CD4^+^ T-cell latency models in which infected activated cells transition toward a quiescent state characterized by reduced proliferation, effector function, and viral transcription [10,11,12]. In cells of the monocyte/macrophage lineage, TGF-β was also shown early in the HIV epidemic to directly suppress HIV expression and replication [13]. More recently, our group demonstrated in vivo that pharmacologic blockade of TGF-β signaling reactivates HIV/SIV reservoirs and enhances antiviral immune responses [14]. In SIV-infected rhesus macaques, TGF-β blockade promoted a shift toward effector-memory T-cell phenotypes and was accompanied by broad metabolic reprogramming [15]. Single-cell analysis subsequently demonstrated that these metabolic changes occurred within SIV-transcribing cells themselves and linked TGF-β signaling to suppression of cellular metabolic activity and viral transcription [16].

These observations suggest that regulation of cellular metabolism may represent an important mechanism through which TGF-β promotes viral quiescence. Cellular metabolism is tightly coupled to immune-cell activation and differentiation [17], and metabolic state has increasingly been recognized as an important determinant of HIV transcription and persistence [18]. Recent studies have further linked HIV-associated quiescence to coordinated suppression of anabolic and energy-generating programs, including glycolysis, oxidative phosphorylation, and mTORC1 signaling [19]. However, the cell-intrinsic mechanisms through which TGF-β induces metabolic suppression and how these changes ultimately constrain HIV transcription remain poorly defined.

To address this question, we used the provirally infected OM10.1 myeloid-cell model [20]. We hypothesized that TGF-β would restrain HIV reactivation by reducing cellular metabolic activity. We found that TGF-β broadly suppressed glycolytic and mitochondrial metabolism and markedly inhibited *c-Myc* signaling. Restoring MYC expression rescued HIV transcription and partially restored viral protein production, identifying *c-Myc*-dependent metabolic reprogramming as an important component of TGF-β-mediated suppression of HIV reactivation.

## 2. Materials and Methods

### 2.1. OM10.1 Reactivation Assays and p24 Intracellular Staining

Cells were cultured for 18 h overnight in RPMI supplemented with 10% FBS and penicillin/streptomycin at a density of 5 × 10^5^ cells per mL. Reactivation was induced using either PMA at 10 ng/mL (Sigma-Aldrich) or recombinant human TNF-α at 5 ng/mL (PeproTech, Cranbury, NJ, USA). Cells were treated with TGF-β at 10 ng/mL (R & D Systems, Minneapolis, MN, USA), Gal at 10 μM (MedChem Express, Monmouth Junction, NJ, USA), a combination of TGF-β and Gal, or left untreated as a mock control. Experiments were performed in 96-well plates with 10^5^ cells per well in technical triplicate or quadruplicate as indicated in the legends. *n* = 4 independent experiments were run for each LRA with treatments of either TGF-β or TGF-β + SB 525334 at 2 μM (MedChem Express, Monmouth Junction, NJ, USA), with untreated cells as a mock control.

Cells were collected, washed, and stained with the fixable viability dye FVS440UV (BD, La Jolla, CA, USA). Cells were then fixed and permeabilized using the BD Cytofix/Cytoperm kit for 30 min at 4 °C. Following fixation, cells were stained with a p24 KC57-PE antibody (NIH HIV Reagent Program, Manassas, VA, USA) for 20 min at room temperature protected from light. Cells were then immediately acquired on a Cytek Aurora 4-laser spectral flow cytometer (Cytek Biosciences, Fremont, CA, USA). Data were analyzed using FlowJo (v10.10.1) to determine the percent of cells expressing detectable p24. Using the mean percent of p24-positive cells from each assay’s mock condition as a baseline, the p24 fold change for each treatment condition was calculated. Fold-change data from each group of LRA experiments was pooled for comparison.

Anti-TNF-α antibody efficacy was evaluated by inducing reactivation in cells and treating with TGF-β, with untreated cells as a control. These two conditions were repeated with the addition of 1 μg/mL of the anti-TNF-α antibody (ThermoFisher, Waltham, MA, USA; concentration selected based on the manufacturer’s validated in vitro neutralization protocol) 1 h after reactivation (*n* = 3 technical replicates). After 17 more hours of incubation, cells were stained for p24. The inclusion of the anti-TNF-α antibody 1 h after reactivation was incorporated into all further experiments for all treatment conditions.

For the BAM15 assay, cells were cultured following the same methods as the TNF-α-reactivated p24 assays. BAM15 (1 µM; MedChem Express, Monmouth Junction, NJ, USA) was added simultaneously with TNF-α and maintained for the full 18 h incubation before p24 staining (*n* = 3 technical replicate wells per condition).

### 2.2. TAR and Gag RT-qPCR

Cells were cultured in a 12-well plate with 6 × 10^5^ cells per well, reactivated with TNF-α, and treated with TGF-β or TGF-β + SB 525334, with untreated and unstimulated groups included as controls (*n* = 3 technical replicates). At both 4 h and 18 h post-reactivation, cell pellets of 2 × 10^5^ cells each were collected and used for total RNA extraction using the RNeasy Mini Kit (Qiagen, Hilden, Germany) with on-column DNase treatment. The remaining 2 × 10^5^ cells were used to perform a confirmatory p24 stain. Equal masses of total RNA from each extraction were used with the SuperScript IV cDNA synthesis kit (Invitrogen, Waltham, MA, USA), and 5 μL of the resultant cDNA from each reaction was mixed with 15 μL of the IDT GeneExpression Master Mix to run each sample in duplicate on an Applied Biosystems QuantStudio 3 (ThermoFisher, Waltham, MA, USA). Due to overlap between the gag and TAR sequences, each reaction was run separately, with quantification for each performed using the standard curve method. TAR-to-gag ratios were determined by calculating the mean of the duplicates from each technical replicate for both TAR and gag and dividing the TAR mean by the gag mean. The following sequences were used as primers and probes:

Gag forward: TACTGACGCTCTCGCACC; Gag reverse: TCTCGACGCAGGACTCG; Gag probe: /FAM/CTCTCTCCT/ZEN/TCTAGCCTC/3IABkFQ/; TAR forward: GTCTCTCTGGTTAGACCAG; TAR reverse: TGGGTTCCCTAGYTAGCC; TAR probe: /FAM/AGCCTGGGA/ZEN/GCTC/3IABkFQ/. qPCR was conducted with an initial hold stage at 50 °C for 15 min, then 95 °C for 3 min. This was followed by 40 amplification cycles of 95 °C for 15 s and 60 °C for 1 min.

### 2.3. Phospho-NF-κB and Phospho-AKT Staining

For NF-κB, cells were plated in a 48-well plate at a density of 2 × 10^5^ cells per well. With unstimulated cells and TNF-α-only cells as controls, TGF-β was given either 1 h before or 1 h after TNF-α stimulation. One-fifth of the cells were collected at 30 min, 45 min, 2 h, and 3 h post-reactivation. The 30 min time point was tested with 6 technical replicates, and the other time points were tested with 3 technical replicates. For each time point, the cells were stained with the fixable viability dye FVS440UV before being washed and fixed using the Invitrogen Fix/Perm kit following the manufacturer’s instructions. The cells were then blocked with mouse IgG (Sigma-Aldrich, Burlington, MA, USA) and stained with an NF-κB p65 antibody (BD, La Jolla, CA, USA) for 45 min at RT in the dark. The cells were then immediately acquired and analyzed via flow as described in the p24 assay. The remaining cells were left to incubate overnight for a confirmatory p24 stain.

For AKT, cells were plated in a 96-well plate at a density of 10^5^ cells per well with unstimulated and TNF-α conditions (*n* = 3 technical replicates). Half of the cells were harvested at 30 min, and the other half were harvested 2 h after TNF-α stimulation. The cells were stained with the fixable viability dye FVS440UV, then washed and fixed using the Invitrogen Fix/Perm kit. The cells were then blocked with mouse IgG and stained with a p-AKT (Ser473) antibody (ThermoFisher, Waltham, MA, USA) for 45 min at RT in the dark. The cells were then immediately acquired and analyzed via flow as described in the p24 assay.

### 2.4. RNA-Sequencing and Analysis

Cells were reactivated with TNF-α and treated with TGF-β, TGF-β + Gal, or TGF-β + SB 525334, with untreated cells as a control in quadruplicate. Cell pellets were harvested at 4 h post-reactivation for RNA extraction. Total RNA was isolated in the same fashion as the qPCR extractions, and RNA quantity and quality were assessed by high-sensitivity RNA electrophoresis at the Northwestern University Metabolomics Core Genomics Branch. Ten nanograms (10 ng) of total RNA were submitted to the core for total RNA library preparation, followed by sequencing using an Illumina NextSeq 2000 (Illumina, San Diego, CA, USA) with single-end reads, resulting in >20 M reads per sample.

FASTQ files for each sample were first assessed using FastQC to verify sequencing quality. Sequence reads were quantified using Salmon (v1.10.3) against the GENCODE v44 human reference transcriptome. The Salmon outputs were imported into R (v4.4.1) using the tximport package (v1.32.0) from Bioconductor (v3.20), converting transcript quantification into gene-level quantification. Variance-stabilizing transformation using DESeq2 was performed, followed by principal component analysis (PCA) clustering and visualization using the ggplot2 package (v4.0.0). Differential gene expression (DEG) analysis was performed using DESeq2 with treatment as a categorical factor, comparing the three treated conditions to the mock condition as a baseline. Statistical significance was defined using the Benjamini–Hochberg false discovery rate (BH-FDR) method, with genes considered significantly differentially expressed at BH-FDR ≤ 0.05 and an |log_2_ fold change| ≥ 1. Volcano plot visualization of DEGs was performed using ggplot2. DEGs were used for further gene set enrichment analysis (GSEA, v4.4.0) using the Hallmark gene sets, with normalized enrichment score (NES) visualization of the top 10 most enriched and depleted pathways.

### 2.5. Agilent Seahorse Assay and TMRM Staining

Cells were incubated overnight at a density of 10^5^ cells per mL, using the same treatment conditions as the p24 assays. Cells were collected and resuspended in Agilent Seahorse XF RPMI media supplemented with 0.2 mM glucose, 2 mM glutamine, and 1 mM pyruvate before being seeded into a Seahorse XFe96 PDL-coated plate at a density of 75,000 live cells per well (cell number and viability were measured after treatment immediately before plating, and an identical input of 75,000 live cells was seeded per well for all conditions). Each treatment was seeded in either *n* = 5 technical replicates (mock) or *n* = 6 technical replicates (treated conditions) before being loaded into an Agilent XFe96 analyzer (Agilent, Santa Clara, CA, USA). Basal O_2_ and pH flux readings were measured, followed by subsequent injections with Oligomycin (1.5 μM) and Rotenone/Antimycin A (0.5 μM) according to Agilent’s ATP Rate Assay protocol. Data were analyzed using the Agilent Seahorse Analytics software (v1.0.0-796).

For TMRM staining, cells were cultured as above, washed and stained with the viability dye FVS440UV, with the remaining cells being used for a confirmatory p24 stain. The cells were washed and stained with FVS440UV viability dye and then stained with the MitoProbe TMRM Assay Kit (ThermoFisher) at a 1:1000 dilution in ice-cold phenol red-free HBSS before being placed into a 37 °C incubator for 30 min. Cells were then spun down and resuspended in FACS buffer and immediately acquired via flow cytometry and analyzed in FlowJo. TMRM fluorescence was quantified within the singlet/live-cell gate with approximately the same number of events acquired across conditions.

### 2.6. Overexpression of MYC and c-Myc/SRM RT-qPCR

Untreated cells were collected and placed into a 16-well electroporation strip at a density of 4 × 10^5^ cells per well using the Lonza SF nucleofection kit. Each well also contained either 0.4 μg of a *c-Myc* plasmid (Addgene 16011) or 0.4 μg of an empty control plasmid (JH038). The cells were then placed in the X-unit of a Lonza 4D-nucleofector and electroporated using the manufacturer’s EN-138 pulse code. Immediately after, the cells were given 100 μL of RPMI supplemented with 20% FBS and placed into a 37 °C incubator for 30 min to recover. Following the recovery period, the cells for each plasmid condition were pooled and transferred to a flask containing RPMI supplemented with 10% FBS to incubate for 48 h at a density of 2 × 10^5^ cells per mL to allow time for the *c-Myc* plasmid to reach maximum expression.

Following incubation, cells were collected and seeded into a 48-well plate at a density of 2 × 10^5^ cells per well in technical quadruplicate for each condition. Non-transfected control and JH038-transfected cells were treated with either TNF-α alone or TNF-α + TGF-β, while Myc-transfected cells were treated with TNF-α + TGF-β only. After 18 h, 10^5^ cells were collected for RNA extraction, and the rest of the cells were used for a p24 stain. The extracted RNA was processed into cDNA and used for qPCR using the same protocol as the previously described gag and TAR qPCR.

For the transfection with lipid nanoparticles, cells were plated into a 96-well plate at 10^5^ cells per well in technical triplicate, stimulated with TNF-α, and treated with TGF-β, TGF-β + 2 μg of LNP-coated *c-Myc* mRNA (ProMab) or TGF-β + 10 μg of LNP-coated *c-Myc* mRNA, with untreated cells as a control. The cells were placed on a shaker set to low speed overnight and left to incubate. After 18 h, the cells were harvested and stained for p24.

To confirm inhibition of *c-Myc* and the MYC target SRM in non-transfected cells, TNF-α-stimulated cells treated with TGF-β ± SB 525334, with untreated cells as a control, were used to harvest RNA following the same protocol previously described for the gag and TAR qPCR. cDNA was generated with SuperScript IV and the cDNA measured by SYBR Green using the ΔΔ*C*_t_ method with GAPDH as the housekeeping gene. The following sequences were used as primers:

*c-Myc* forward: ACGAAACTTTGCCCATAGCA; *c-Myc* reverse: GCAAGGAGAGCCTTTCAGAG; SRM forward: CCCTCCGTGGAGTCCGTGGTC; SRM reverse: CTGGCAGGAACTTCTTGGAGACTTG GAPDH forward: GAAGGTGAAGGTCGGAGTC; GAPDH reverse: GAAGATGGTGATGGGATTTC. qPCR was conducted with an initial hold stage at 50 °C for 15 min, then 95 °C for 3 min. This was followed by 40 amplification cycles of 95 °C for 15 s and 60 °C for 1 min.

### 2.7. Surface Marker Staining

Cells were seeded into a 96-well plate with 10^5^ cells per well in technical triplicate, reactivated with TNF-α, and treated with TGF-β, Gal, or a combination of both, with untreated cells as a control. After 18 h of incubation, the cells were collected and stained with fixable viability dye FVS440UV. They were then washed and blocked with mouse IgG before staining with CD11b (Biolegend, San Diego, CA, USA), CD25 (Biolegend, San Diego, CA, USA), HLA-DR (Biolegend, San Diego, CA, USA), CD80 (BD, La Jolla, CA, USA), CD86 (Biolegend, San Diego, CA, USA), and CD163 (BD, La Jolla, CA, USA) in FACS buffer for 20 min at 4 °C. The cells were then fixed with the BD perm/fix kit and stained for p24, followed by immediate acquisition and analysis as described in the p24 assay.

### 2.8. Statistical Analysis

Statistical analysis was performed using GraphPad Prism (v11.0.2) and R (v4.4.1). Excluding the RNA-sequencing, all experiments were analyzed using two-way ANOVA with Tukey’s Honestly Significant Difference test to correct for multiple comparisons. Statistical significance of individual comparisons is denoted throughout as * *p* ≤ 0.05, ** *p* ≤ 0.01, *** *p* ≤ 0.001, and **** *p* ≤ 0.0001. For the RNA-sequencing data, comparisons were made using the Benjamini–Hochberg false discovery rate (BH-FDR) method, and significantly differentially expressed genes were defined as having a BH-FDR ≤ 0.05 and an |log_2_ fold change| ≥ 1. For GSEA analysis of the RNA-sequencing data, comparisons were made using FDR-q values for the enriched pathways, displayed as * q ≤ 0.05, ** q ≤ 0.01, *** q ≤ 0.001, and **** q ≤ 0.0001.

## 3. Results

### 3.1. TGF-β Inhibits Proviral Reactivation in OM10.1 Cells

To determine whether TGF-β restrains viral reactivation in OM10.1 cells, we stimulated cells for 18 h with one of two latency reversal agents (LRAs): phorbol 12-myristate 13-acetate (PMA) [21], a protein kinase C agonist, or tumor necrosis factor alpha (TNF-α) [22], an NF-κB agonist. For each LRA, cells were assigned to four treatment groups: LRA alone, LRA plus exogenous TGF-β, LRA plus the TGF-β receptor I (TGFβRI/ALK5) inhibitors galunisertib (Gal, IC_50_ 56 nM) [23] or SB 525334 (SB, IC_50_ 14.3 nM) [24], or LRA plus both TGF-β and galunisertib.

**Figure 1:**
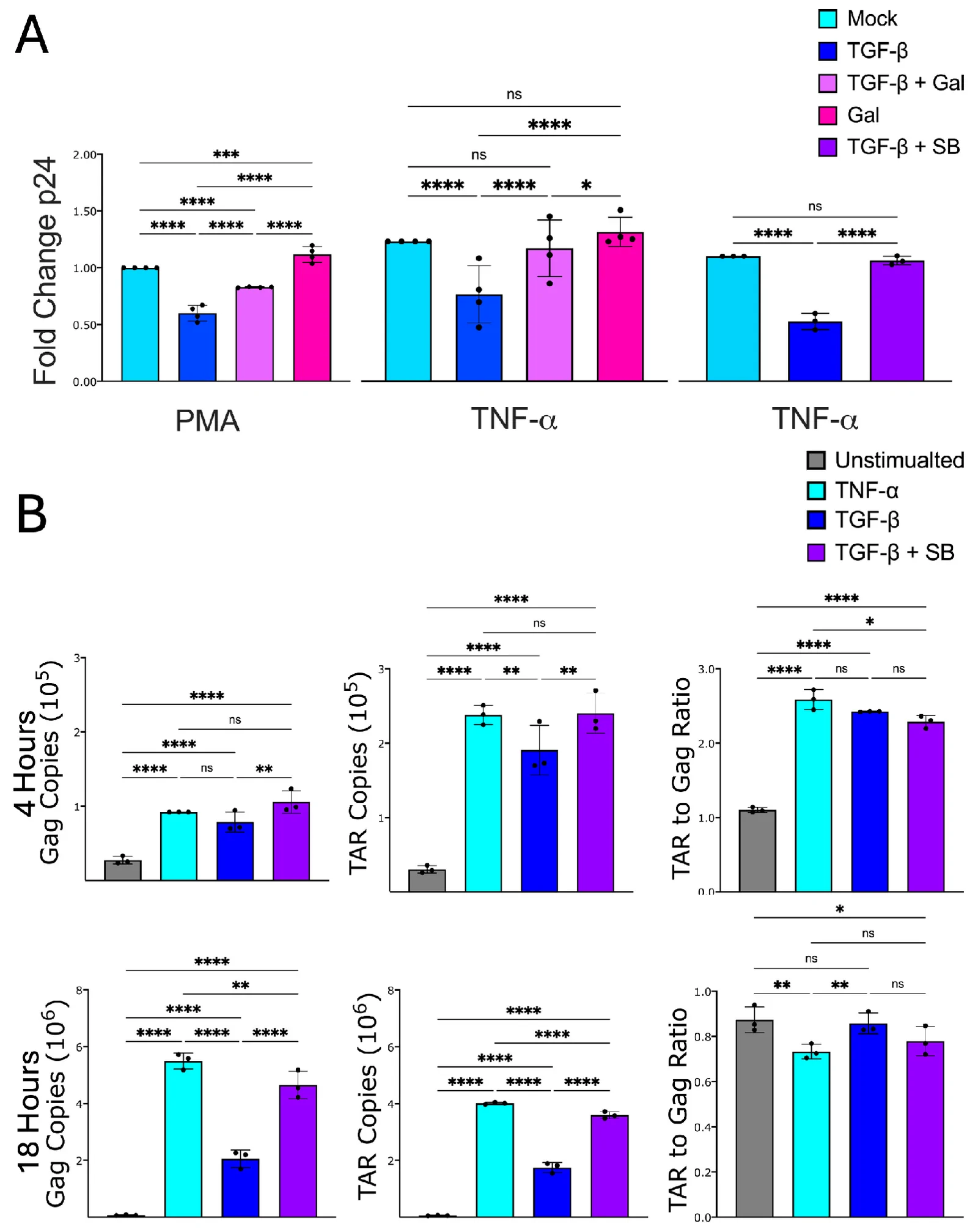
TGF-β inhibits latency reversal in OM10.1 cells. (**A**) Pooled fold-change in p24+ cells frequencies (gated on singlets, live cells) for PMA-stimulated (*n* = 4 independent experiments) and TNF-α-stimulated (*n* = 4 independent experiments) OM10.1 cells treated with TGF-β, Gal, or both, and TNF-α-stimulated cells treated with TGF-β ± SB525334 (*n* = 3 independent experiments). Each independent experiment was performed in technical triplicate; within each experiment, the mean frequency of p24^+^ cells in the mock (LRA-only) condition served as the baseline for calculating fold change. Fold-change values were pooled across independent experiments and compared by two-way ANOVA with Tukey’s correction for multiple comparisons. (**B**) qPCR quantification of gag and TAR transcripts from cDNA synthesized from OM10.1 cells stimulated with TNF-α and treated with TGF-β or TGF-β + SB525334, with unstimulated cells as a baseline control. Four technical replicates per condition, and cDNA was generated from equal masses of total input RNA. gag copies were compared directly with TAR copies per technical replicate to determine the TAR:Gag ratio. One of two independent experiments with similar data is shown. Data are shown at 4 and 18 h post-reactivation and were compared by two-way ANOVA with Tukey’s correction. * *p* ≤ 0.05, ** *p* ≤ 0.01, *** *p* ≤ 0.001, **** *p* ≤ 0.0001.

Using the LRA-only condition as the within-experiment baseline, we calculated the fold change in the frequency of p24^+^ cells for each condition and pooled these values across four independent experiments (Figure 1A and Figure S1). TGF-β markedly reduced p24 production under both LRAs, decreasing the frequency of p24^+^ cells by ~40.0% relative to baseline, while co-treatment with galunisertib or SB 525334 reversed this effect. Galunisertib alone modestly enhanced reactivation, consistent with relief of tonic endogenous TGF-β signaling, as we previously showed [14].

In order to test a more physiologically relevant LRA, we conducted the remaining experiments with TNF-α, an inflammatory cytokine known to reactivate viral transcription, particularly in myeloid cells [25]. TNF-α does, however, drive a positive autocrine feedback loop [26], whereby exogenous TNF-α amplifies endogenous TNF-α production and could obscure the effect of TGF-β. To control for this, we added a neutralizing anti-TNF-α antibody one hour after the initial TNF-α stimulation. Neutralization of endogenously released TNF-α had a small inhibitory effect on HIV reactivation, but it enhanced the inhibitory effect of TGF-β (Figure S2). We therefore included the anti-TNF-α antibody in all subsequent experiments.

To complement the p24 protein measurements and to determine whether the TGF-β inhibition was at the level of transcriptional initiation or elongation, we assessed the effect of TGF-β on TAR and Gag transcripts. Cells were stimulated with TNF-α and treated with TGF-β or TGF-β + SB 525334 and harvested at 4 and 18 h. At 4 h, TNF-α, as expected, substantially increased both TAR and Gag transcripts, with a larger increase in TAR and TAR:Gag ratio (Figure 1B).

TGF-β did not significantly alter Gag transcripts but modestly reduced TAR transcripts relative to TNF-α alone and the TGF-β + SB 525334 control, but the TAR:Gag ratio remained substantially unchanged at this time point. By 18 h, TGF-β sharply reduced both Gag and TAR transcripts relative to TNF-α alone, accompanied by a small but significant increase in the TAR:Gag ratio (Figure 1B). Although the TAR:Gag ratio did not differ significantly between TGF-β and TGF-β + SB525334, the significant increase in the ratio between the mock and TGF-β conditions suggests that TGF-β may have an additional effect on transcriptional elongation. Together, these data demonstrate that TGF-β suppresses HIV transcription in OM10.1 cells, with a possible additional effect on transcriptional elongation.

### 3.2. TGF-β Suppresses c-Myc Signaling and Metabolic Pathways

We next sought the mechanism underlying this inhibition. Because TGF-β appeared to act at both the initiation and elongation stages of HIV transcription, we examined the two pathways that govern these steps during TNF-α-driven reactivation: NF-κB [27], which binds the κB enhancer elements in the 5′ LTR to drive transcriptional initiation, and the AKT [28] pathway, which acts indirectly to promote elongation. The levels of phospho-NF-κB p65 (Ser529), as expected, increased 30 min after TNF-α addition relative to unstimulated cells, but this increase was only slightly further increased by TGF-β given before or after the TNF-α and phospho-p65 levels across all groups converged with the unstimulated control by 2 h (Figure S3A). The levels of phospho-AKT (Ser 473) did not differ between unstimulated and TNF-α-stimulated cells (Figure S3B), indicating that AKT signaling is not a major driver of TNF-α-induced latency reversal in OM10.1 cells. Thus, TGF-β-mediated inhibition of reactivation does not appear to act through suppression of the NF-κB or AKT pathways.

Nevertheless, TGF-β induced substantial phenotypic remodeling of OM10.1 cells. TGF-β reduced expression of CD11b and CD25 while increasing HLA-DR, CD80, and CD86. Most strikingly, TGF-β strongly induced CD163, a marker associated with homeostatic and anti-inflammatory macrophage states [29], suggesting remodeling toward a macrophage-like phenotype with immunoregulatory features (Figure S4). Notably, tissue-conditioned intestinal macrophages can acquire an immunoregulatory phenotype with reduced permissiveness to HIV replication, and TGF-β has been implicated in establishing this state [30]. Thus, the phenotypic remodeling observed here could itself contribute to reduced viral reactivation; however, our data do not distinguish this effect from the concurrent metabolic suppression induced by TGF-β

**Figure 2:**
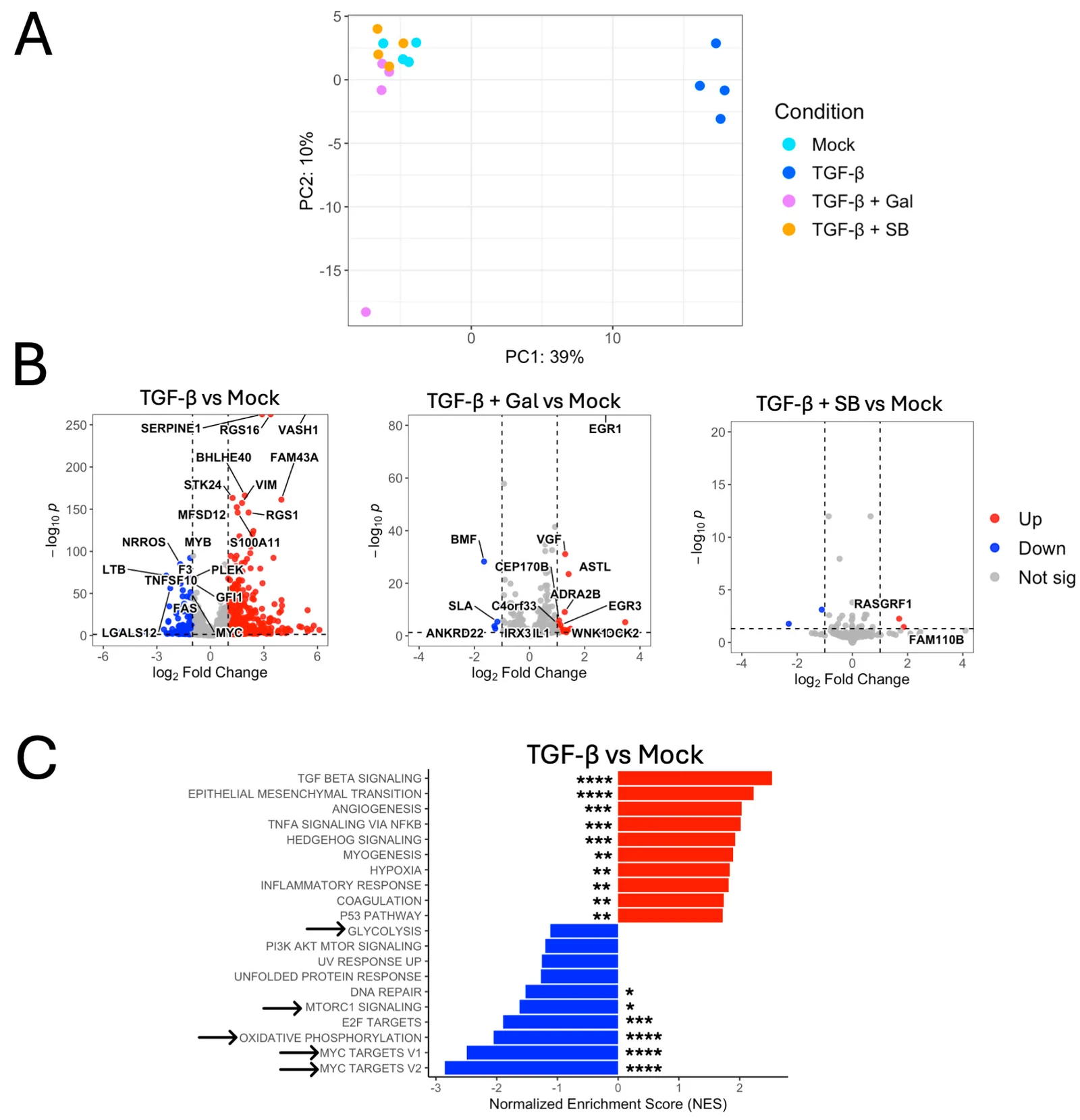
TGF-β suppresses *c-Myc* signaling and metabolic pathways. (**A**) PCA of bulk RNA-seq samples. OM10.1 cells were stimulated with TNF-α and treated with TGF-β, TGF-β + Gal, or TGF-β + SB 525334, in technical quadruplicate, before RNA extraction. Libraries were sequenced on an Illumina NextSeq 2000 in single-read format, and PCA was performed after variance-stabilizing transformation (DESeq2). (**B**) Volcano plots of DEGs for each treatment group relative to the mock (TNF-α only) control, computed with DESeq2. Significance was defined as FDR ≤ 0.05 and |log_2_ fold change| ≥ 1 (cutoffs displayed as dashed lines). The ten most significant protein-coding up- and downregulated genes are labeled. (**C**) GSEA of DEGs from TGF-β-treated cells relative to the mock control, using the Hallmark gene set. The ten most enriched and depleted pathways by NES are shown; arrows indicate metabolic pathways of interest. FDR-q values for the enriched pathways are displayed. * q ≤ 0.05, ** q ≤ 0.01, *** q ≤ 0.001, **** q ≤ 0.0001.

Given these broad phenotypic effects, we performed bulk RNA sequencing to define the transcriptional programs altered by TGF-β. OM10.1 cells were stimulated with TNF-α and treated with TGF-β, TGF-β + Gal, or TGF-β + SB 525334, in technical quadruplicate, and RNA was extracted 4 h after stimulation. Following variance-stabilizing transformation, principal component analysis (PCA) showed that the mock (TNF-α only), TGF-β + Gal, and TGF-β + SB 525334 conditions formed a single tight cluster, while the TGF-β condition separated distinctly along PC1 (Figure 2A). One TGF-β + Gal replicate mapped away from the main cluster along PC2, which accounted for only 10% of the total variance, and was therefore retained in downstream analyses.

We then examined differentially expressed genes (DEGs) relative to the mock (TNF-α only) baseline using volcano plots (Figure 2B), defining significant DEGs as those with a false discovery rate (FDR) ≤ 0.05 and a log_2_ fold change ≥ 1 in absolute value. The most upregulated genes reflected previously described TGF-β-inducible programs [31,32,33], including epithelial-to-mesenchymal transition (VIM [34], S100A11 [35], hypoxia-inducible HIF-1α signaling (SERPINE1 [36], BHLHE40/DEC1 [37]), and immunosuppression (RGS16 [38], RGS1 [39]). The most downregulated genes were associated with metabolic regulation (LGALS12/Galectin-12 [40], MYC [41]), immune-cell maturation and differentiation (GFI1 [42], MYB [43]), and apoptosis (FAS [44], TNFSF10/TRAIL [45]), all previously linked to TGF-β [46,47,48]. Consistent with target engagement, galunisertib curtailed the TGF-β transcriptional response (only 14 significant DEGs compared to mock), and SB 525334, with its lower IC_50_ for ALK5, restrained it further (only four significant DEGs compared to mock).

To identify the pathways most affected by TGF-β, we performed gene set enrichment analysis (GSEA [49]) on the ranked gene list using the Hallmark gene sets [50] (Figure 2C). TGF-β enriched TGF-β signaling (NES = 2.53), epithelial-to-mesenchymal transition (NES = 2.23), and hypoxia (NES = 1.84), mirroring the most upregulated individual genes. Conversely, TGF-β depleted *c-Myc* Targets V2 and V1 (NES = −2.85 and −2.50), MTORC1 signaling (NES = −1.62), oxidative phosphorylation (NES = −2.05), and glycolysis (NES = −1.11). The coordinated downregulation of these anabolic and energy-generating pathways pointed to a global reduction in metabolic activity, plausibly driven by *c-Myc* suppression given the central role of *c-Myc* in controlling cellular metabolism [41,51]. Consistent with this, TGF-β-mediated suppression of c-*MYC* and the MYC target gene *SRM* was confirmed by RT-qPCR (Figure S5). Together, these data support our hypothesis that TGF-β-driven suppression of HIV transcription is linked to reduced cellular metabolism, and they implicate *c-Myc* as a likely mediator of this effect.

### 3.3. TGF-β Impairs Metabolic Function and Mitochondrial Polarization

**Figure 3:**
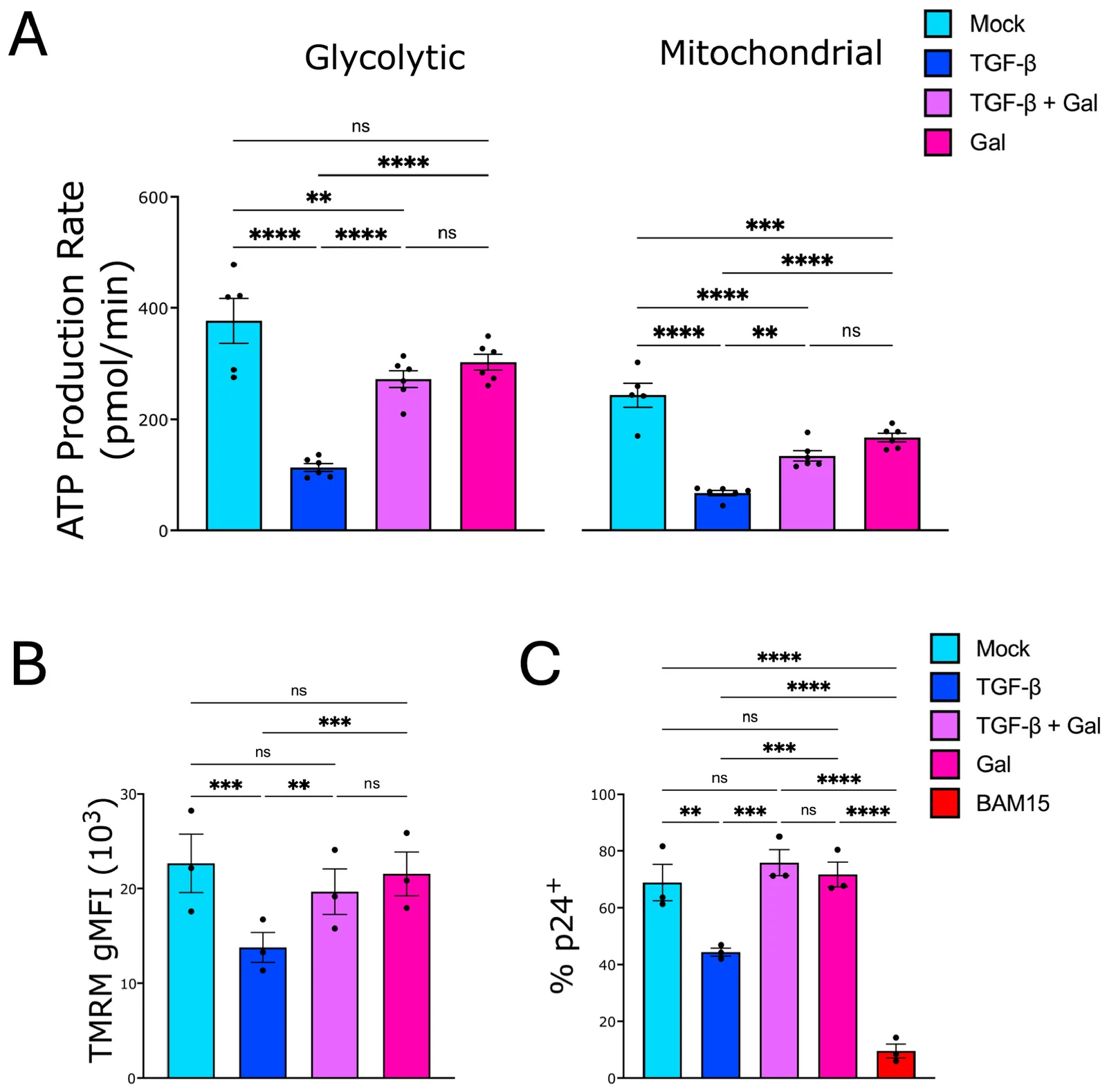
TGF-β impairs metabolic function and mitochondrial polarization. (**A**) Glycolytic and mitochondrial ATP production rates in TNF-α-stimulated OM10.1 cells treated with TGF-β, Gal, or both, measured on a Seahorse XFe96 analyzer with the XF Real-Time ATP Rate Assay. The mock condition was plated in five technical replicate wells and treated conditions in six technical replicate wells; all wells were seeded with 75,000 viable cells following post-treatment cell counting. ATP production rates were derived using Agilent Seahorse Analytics software (v1.0.0-796). One of three independent experiments with similar results is shown. (**B**) TMRM staining of mitochondrial membrane potential in unstimulated and TNF-α-stimulated OM10.1 cells treated with TGF-β, Gal, or both, measured by flow cytometry (*n* = 3 technical replicates per condition). TMRM fluorescence was quantified within the singlet/live-cell gate with approximately the same number of events acquired across conditions. One of three independent experiments with similar results is shown. (**C**) Frequency of singlets, live, p24^+^ cells among TNF-α-stimulated OM10.1 cells treated with TGF-β, Gal, TGF-β + Gal, or the mitochondrial uncoupler BAM15 (*n* = 3 technical replicates per condition). One of two independent experiments with similar results is shown. All comparisons were made by two-way ANOVA with Tukey’s correction. ** *p* ≤ 0.01, *** *p* ≤ 0.001, **** *p* ≤ 0.0001.

To test the impact of TGF-β on the functional metabolic output of OM10.1 cells, we used a Seahorse XFe96 analyzer and quantified basal glycolytic and mitochondrial ATP production in TNF-α-reactivated cells treated in the presence of TGF-β, Gal, or both (Figure 3A). TGF-β strongly reduced both glycolytic and mitochondrial ATP production relative to TNF-α alone. Co-treatment with galunisertib largely rescued ATP production, although galunisertib alone was partially inhibitory of mitochondrial ATP.

To corroborate the effect of TGF-β on mitochondrial function, we measured mitochondrial membrane potential using tetramethylrhodamine methyl ester (TMRM) [52] by flow cytometry under the same conditions (Figure 3B). TGF-β reduced membrane potential relative to TNF-α alone, and this effect was blocked by galunisertib. Finally, to test whether impaired metabolism is sufficient to reduce viral reactivation, we repeated the p24 assay with the addition of BAM15, a potent mitochondrial uncoupler that collapses the proton gradient and abolishes mitochondrial ATP production [53] (Figure 3C). BAM15 profoundly suppressed reactivation relative to TNF-α alone without significantly affecting cell viability (Figure S6). Thus, acute mitochondrial uncoupling can independently suppress HIV reactivation in this system, supporting a functional link between metabolic capacity and viral reactivation.

### 3.4. MYC Overexpression Rescues TGF-β-Mediated Inhibition of Latency Reversal

**Figure 4:**
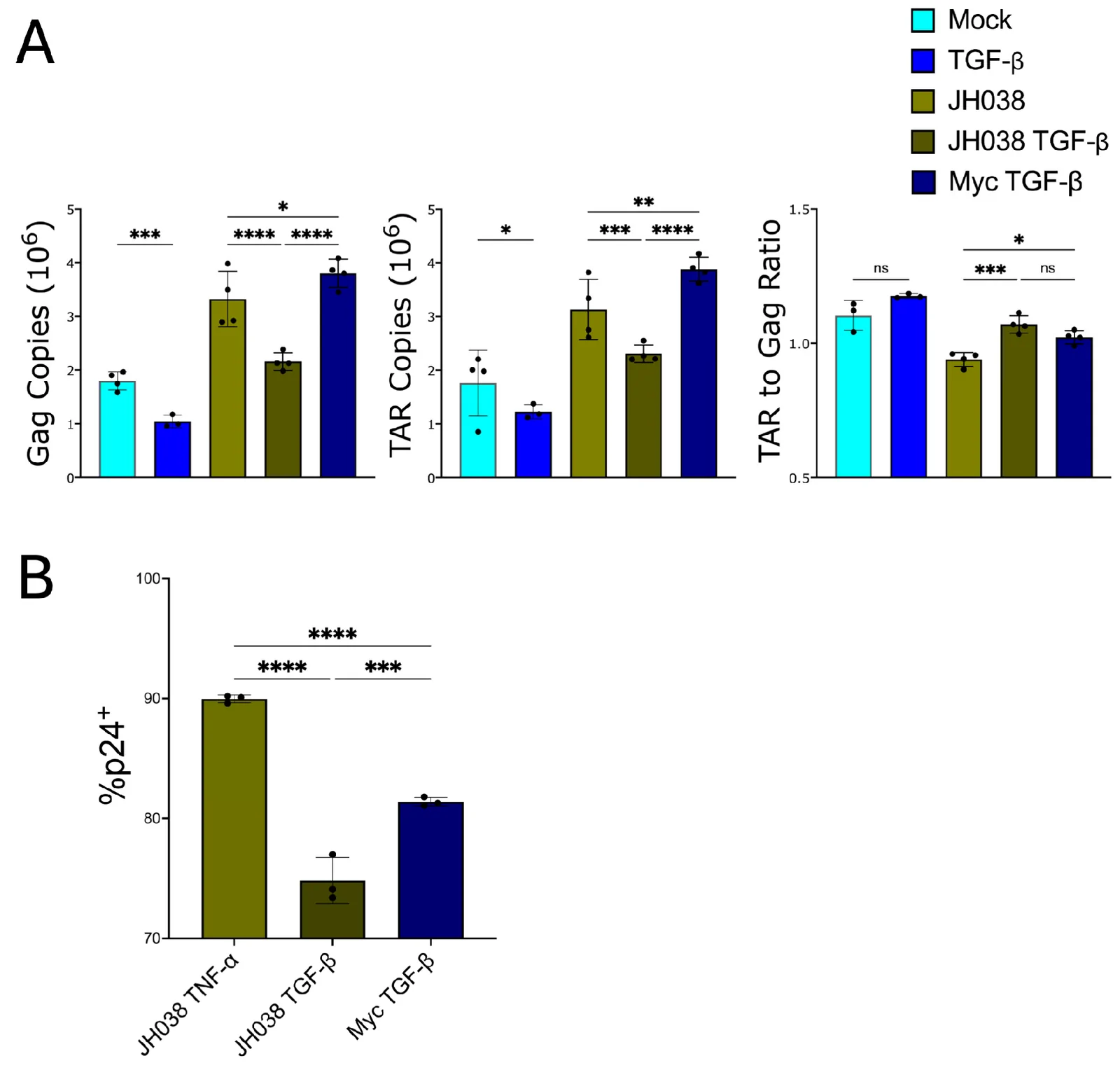
MYC overexpression rescues TGF-β-mediated inhibition of latency reversal. (**A**) Quantification of gag and TAR transcripts from OM10.1 cells stimulated with TNF-α ± TGF-β. Cells were transfected by electroporation with an empty control plasmid (JH038) or a *c-Myc* plasmid (Myc), with non-transfected cells as an additional control. Four technical replicates per condition were run, and two independent experiments were performed with similar results. The TAR:Gag ratio was determined by directly comparing results from technical replicates. (**B**) Frequency of p24^+^ cells after transfection with the JH038 or *c-Myc* plasmid and stimulation with TNF-α ± TGF-β (*n* = 3 technical replicates per condition; one of two independent experiments with similar results is shown). All comparisons were made by two-way ANOVA with Tukey’s correction. * *p* ≤ 0.05, ** *p* ≤ 0.01, *** *p* ≤ 0.001, **** *p* ≤ 0.0001.

To test whether *c-Myc* is a critical mediator of the effects of TGF-β on viral reactivation, we assessed the impact of overexpressing MYC in TGF-β-treated cells. OM10.1 cells were transfected with either an empty control plasmid (JH038) or a *c-Myc*-expressing plasmid (Myc), then cultured overnight with TNF-α ± TGF-β. Cell pellets were harvested at 18 h in technical quadruplicates to quantify gag and TAR transcript levels (Figure 4A). Electroporation itself was stimulatory: JH038 + TNF-α yielded higher gag and TAR levels than non-transfected TNF-α controls, indicating that transfection enhanced TNF-α-induced reactivation. TGF-β reduced both gag and TAR transcripts in control-plasmid-transfected cells. In contrast, MYC overexpression in the presence of TGF-β restored gag and TAR transcript levels to or above those observed in control-plasmid-transfected cells stimulated with TNF-α alone, indicating that MYC overexpression is sufficient to overcome TGF-β-mediated suppression of HIV transcription. As noted above, TGF-β also modestly increased the TAR:Gag ratio in control-plasmid-transfected cells; MYC overexpression shifted this ratio toward that observed in control-plasmid-transfected cells stimulated with TNF-α alone (Figure 4A).

At the viral protein level, MYC overexpression only partially restored the latency-reversal activity of TNF-α in the presence of TGF-β (Figure 4B), a result we confirmed using LNP-delivered *c-Myc* mRNA in place of plasmid electroporation (Figure S7). Thus, whereas MYC overexpression restored HIV transcription, the incomplete rescue of p24 production suggests that TGF-β exerts additional effects downstream of transcription that are not fully overcome by MYC overexpression.

## 4. Discussion

TGF-β has been implicated in the suppression of HIV/SIV reactivation in cell-line and primary-cell models in vitro [13,14] and in SIV-infected macaques in vivo [14,15,16]. Our previous studies demonstrated that blockade of TGF-β signaling promotes viral reactivation in vivo and is accompanied by broad metabolic reprogramming of reservoir-containing cells [15,16]. Here, we extend those observations by defining a cell-intrinsic mechanism through which TGF-β can restrain HIV reactivation. In OM10.1 cells, TGF-β reduced the response to reactivation stimuli and was associated with coordinated suppression of glycolysis, OXPHOS, mTORC1 and *c-Myc* signaling. These transcriptional changes were accompanied by reductions in both glycolytic and mitochondrial ATP production and mitochondrial membrane polarization. Most importantly, restoring MYC expression overcame TGF-β-mediated suppression of HIV transcription and partially restored viral protein production. Together, these findings support a model in which TGF-β limits HIV reactivation, at least in part, by imposing a MYC-low, metabolically quiescent cellular state.

The ability of TGF-β to suppress reactivation induced by distinct stimuli suggested that its effect was not restricted to a single latency-reversing pathway. Consistent with this, reduced response to TNF-α could not be explained simply by failure to activate its major upstream signaling pathway. Instead, these findings suggested that TGF-β altered the cellular state required to support efficient proviral reactivation, prompting us to investigate broader transcriptional changes induced by TGF-β.

Unbiased transcriptomics pointed to TGF-β impacting several metabolic pathways with particularly strong suppression of *c-Myc*-associated transcriptional programs. Functional metabolic measurements closely paralleled these transcriptional changes, demonstrating marked reductions in both glycolytic and mitochondrial ATP production. Moreover, independently disrupting mitochondrial function with BAM15 strongly inhibited viral reactivation, providing functional evidence that metabolic capacity itself contributes to efficient HIV expression. These observations are consistent with our previous in vivo studies, in which TGF-β blockade induced metabolic activation together with SIV reactivation [15,16] and with a broader literature linking reduced cellular metabolism to HIV transcriptional quiescence [54,55]. Of note, recent work in primary CD4^+^ T cells has shown that HIV-driven entry into quiescence is accompanied by coordinated suppression of *c-Myc* and mTORC1 signaling, glycolysis and oxidative phosphorylation [19]. Although these studies involve different cellular systems, their convergence suggests that suppression of the metabolic programs required to support cellular activation may represent a broader mechanism favoring HIV transcriptional quiescence.

A notable finding of our study was the identification of *c-Myc* as a potential mediator linking TGF-β signaling to this metabolically quiescent state. *c-Myc* is a central regulator of cellular growth and metabolism, coordinating glycolysis, oxidative phosphorylation, glutaminolysis and other anabolic programs [51,56]. Importantly, there is a well-established mechanistic precedent for direct repression of *c-MYC* by TGF-β: canonical TGF-β signaling can recruit a Smad3/E2F4/5/p107 repressor complex to the *c-MYC* promoter, resulting in transcriptional repression [57]. Conversely, a recent study identified *c-Myc* suppression as a central component of HIV-induced quiescence in primary CD4^+^ T cells [19]. Our findings connect these previously separate observations by identifying TGF-β as an extracellular signal capable of coupling *c-Myc* suppression to metabolic inhibition and reduced HIV reactivation.

The functional rescue experiments further support this connection. Despite continued exposure to TGF-β, MYC overexpression restored *gag* and TAR transcript levels to or above those observed in control-transfected cells stimulated with TNF-α alone. Thus, restoring MYC is sufficient to overcome the transcriptional block imposed by TGF-β in this system. At the protein level, however, MYC overexpression only partially restored p24 production, an effect reproduced using LNP-delivered Myc mRNA. This difference suggests that the effects of TGF-β are not limited to *c-Myc*-dependent control of HIV transcription and that additional post-transcriptional constraints on viral production are not fully overcome by *c-Myc* restoration. These additional post-transcriptional mechanisms were not dissected in the present study and will require further investigation.

Our transcriptional measurements also raise the possibility that *c-Myc* may affect HIV expression through mechanisms beyond its broad control of cellular metabolism. TGF-β produced a modest increase in the TAR:Gag ratio, raising the possibility of an additional effect on transcriptional elongation. Importantly, the magnitude of this shift was similar in non-electroporated cells and control-plasmid-transfected cells, despite the overall increase in HIV transcription associated with electroporation, whereas MYC overexpression reduced the shift. MYC has previously been shown to recruit P-TEFb to transcriptional complexes [58], providing a potential mechanistic link between reduced MYC expression and impaired transcriptional elongation. Given the central role of P-TEFb in Tat-dependent HIV transcription [59], altered P-TEFb recruitment or activity could therefore contribute to the effect of TGF-β independently of, or in addition to, global metabolic suppression. This possibility is not directly addressed by the current experiments and should be investigated in future work.

Several limitations should be considered. OM10.1 is an HL-60-derived promyelocytic cell line and therefore does not fully reproduce the biology of primary HIV-infected myeloid cells. HL-60 cells also have dysregulated *c-Myc* expression [60], which could increase their dependence on *c-Myc* and amplify the effects observed here. Finally, although MYC overexpression functionally rescued HIV transcription, we were unable to directly determine whether it also reverses the TGF-β-induced metabolic phenotype because electroporation itself substantially perturbs cellular activation and metabolism. Thus, the present data establish a functional connection between TGF-β, *c-Myc* and HIV transcription but do not yet demonstrate that restoration of metabolism is the direct intermediate through which MYC rescues viral reactivation. Addressing this question using less perturbing approaches and extending these findings to primary cells will be important next steps.

Overall, our findings identify suppression of *c-Myc* and cellular metabolism as a cell-intrinsic mechanism through which TGF-β limits HIV reactivation. They provide a mechanistic framework linking our previous in vivo observations of TGF-β-dependent metabolic quiescence to direct regulation of viral transcription and place *c-Myc* at the intersection of TGF-β signaling, cellular metabolic state and HIV reactivation.

## Data Availability

All relevant data are included in the manuscript, extended data files and Supplemental Material. Values for all data points in graphs are reported in the Source Data file. Raw data files are available to be shared upon request to the corresponding author. All RNA sequencing data originating from this study have been deposited in NCBI GEO under the accession code GSE344570.

## Supplementary Materials

The following supporting information can be downloaded at https://www.mdpi.com/article/10.3390/microorganisms14102368/s1, Figure S1: Gating strategy for p24 flow cytometry. Example of the p24+ gating used in the OM10.1 reactivation experiments. Both TNF-⍺ stimulated and unstimulated conditions from the same experiment are shown. Cells were first gated for live cells and singlets before being gated for p24. Figure S2: Anti-TNF-⍺ antibody limits TNF-⍺ positive feedback loop. p24 data from cells stimulated with TNF-⍺ ± TGF-β, with some cells being treated with an anti-TNF-⍺ functional antibody one hour after TNF-⍺ stimulation. Each condition was run in technical triplicate, and data was compared using two-way ANOVA with Tukey’s correction for multiple comparisons (* *p* < 0.05, ** *p* < 0.01, **** *p* < 0.0001). Figure S3: TGF-β inhibits latency reversal irrespective of dosage timing and without inhibiting classical reactivation pathways. (A) Flow cytometry data for NF-kB p65 where OM10.1 cells were stimulated with TNF-⍺ and treated with TGF-β either 1 h before or 1 h after reactivation, with unstimulated cells as a baseline control. Each condition was run in technical triplicate, and readings were taken at 30 min, 45 min, 2 h, and 3 h post-stimulation. Comparisons were made using two-way ANOVA with Tukey’s correction for multiple comparisons (* *p* < 0.05, ** *p* < 0.01, *** *p* < 0.001). (B) Flow cytometry data for p-AKT from OM10.1 cells ± TNF-⍺ stimulation. Each condition was run in technical triplicate, and readings were taken at 30 min and 2 h post-stimulation. Figure S4: TGF-β alters markers of myeloid maturation and APC function. OM10.1 cells were stimulated with TNF-⍺ and then treated with TGF-β or TGF-β + SB 525334 before being stained for common markers of myeloid activation and function as read by flow cytometry. Each condition was run in technical triplicate, and the data was compared using two-way ANOVA with Tukey’s correction for multiple comparisons (** *p* < 0.01, **** *p* < 0.0001). Figure S5: TGF-β decreases transcripts linked to the *c-Myc* target pathway. qPCR data generated from cDNA synthesized from RNA isolated from OM10.1 cells stimulated with TNF-⍺ and treated with either TGF-β or TGF-β + SB525334, with each condition run in either technical triplicates (Myc) or quadruplicates (SRM). Transcripts were analyzed using the ∆∆Ct SybrGreen method with the TNF-⍺ only condition being used as the fold change baseline. GAPDH was used as the housekeeping gene for ∆∆Ct analysis. Comparisons were made using two-way ANOVA with Tukey’s correction for multiple comparisons (* *p* < 0.05, ** *p* < 0.01, **** *p* < 0.0001). Figure S6: Reactivation treatment conditions do not alter cell viability. FVS440UV viability dye flow cytometry data from TNF-α-stimulated OM10.1 cells treated with TGF-β, Gal, TGF-β + Gal, or the mitochondrial uncoupler BAM15 (*n* = 3 technical replicates per condition). All comparisons were made by two-way ANOVA with Tukey’s correction. Figure S7: LNP transfection of *c-Myc*. p24 flow cytometry data from cells transfected via LNP coated *c-Myc* mRNA, using two different LNP concentrations. The cells were stimulated with TNF-⍺ and TGF-β, with non-transfected cells stimulated with TNF-⍺ ± TGF-β as a control. Each condition was run in technical triplicate. Statistical comparisons were made using two-way ANOVA with Tukey’s correction for multiple comparisons (* *p* < 0.05, ** *p* < 0.01, **** *p* < 0.0001).
