# Supplementary material for: c-Myc Links TGF-β Signaling to Metabolic Control of HIV Reactivation in OM10.1 Cells
Source: Microorganisms. 2026 Oct 16;14(10):2368. doi: 10.3390/microorganisms14102368 (PMC13707392; doi:10.3390/microorganisms14102368)
Supplement: Supplementary file 1 [file microorganisms-14-02368-s001.zip › microorganisms_4552847_Supplemental_Figures_Resubmission.pdf]

metabolites

MDPI

Article - Supplemental Materials

c-Myc Links TGF-β Signaling to Metabolic Control of HIV Reactivation in OM10.1 Cells

Jakob Harrison-Gleason, Jinhee Kim, and Elena Martinelli *

Department of Medicine, Division of Infectious Diseases, Feinberg School of Medicine, Northwestern University, Chicago, IL, USA
* Correspondence: elenamartinelli@northwestern.edu

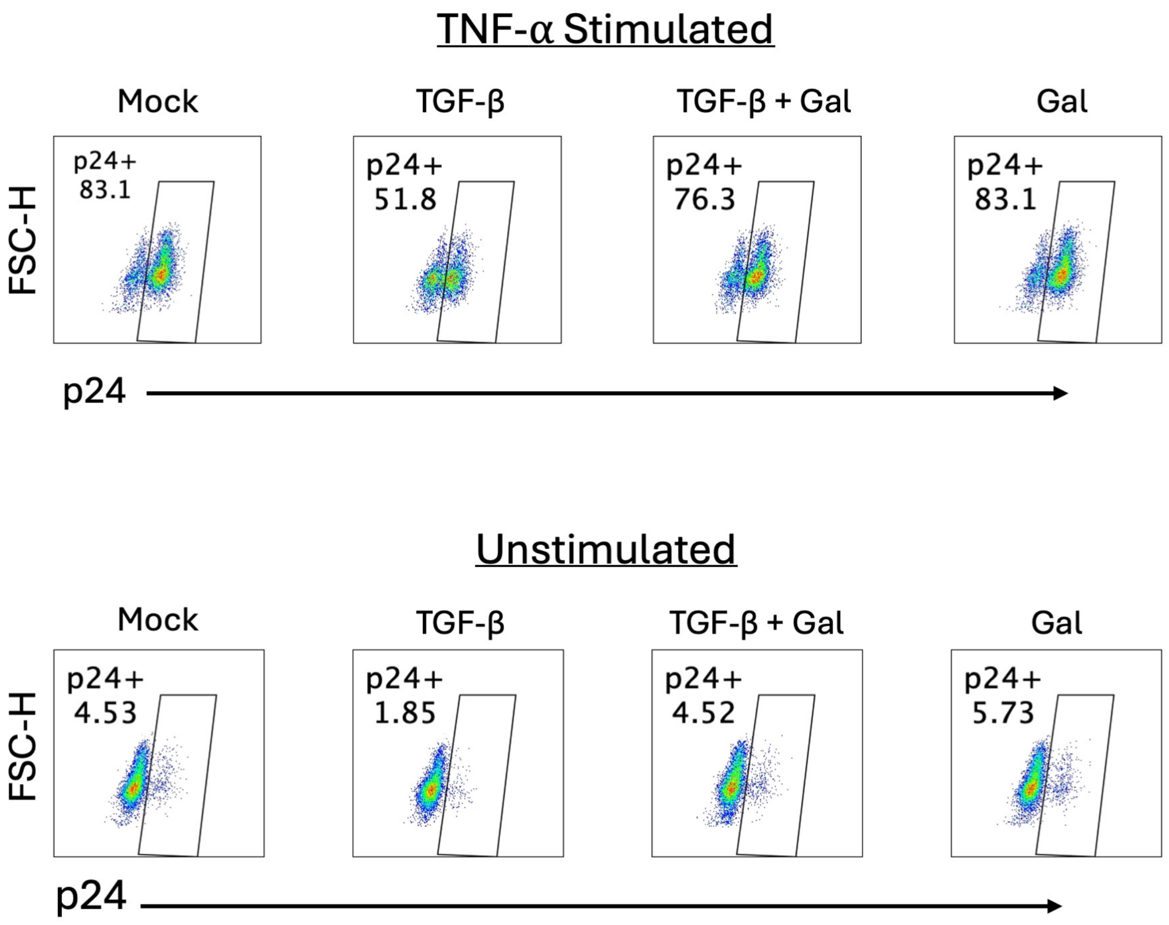

Supplemental Figure S1. Gating strategy for p24 flow cytometry

Example of the p24+ gating used in the OM10.1 reactivation experiments. Both TNF-α stimulated and unstimulated conditions from the same experiment are shown. Cells were first gated for live cells and singlets before being gated for p24

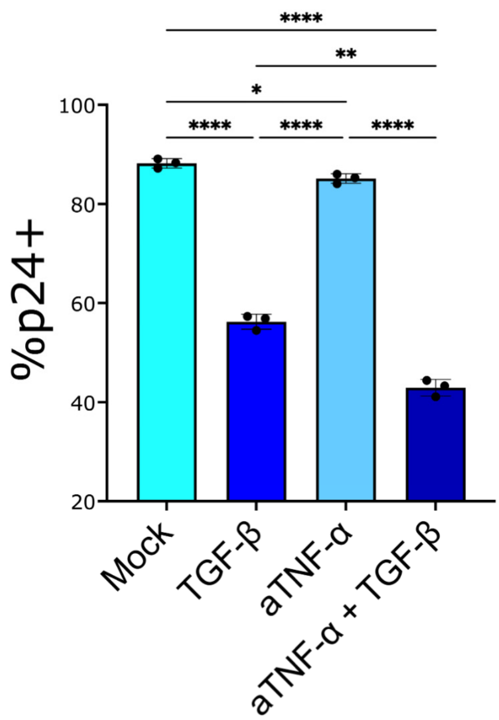

Supplemental Figure S2. Anti TNF-α antibody limits TNF-α positive feedback loop

p24 data from cells stimulated with TNF-α ± TGF-β, with some cells being treated with an anti-TNF-α functional antibody one hour after TNF-α stimulation. Each condition was run in technical triplicate, and data was compared using two-way ANOVA with Tukey's correction for multiple comparisons (*$p < 0.05$, **$p < 0.01$, ****$p < 0.0001$)

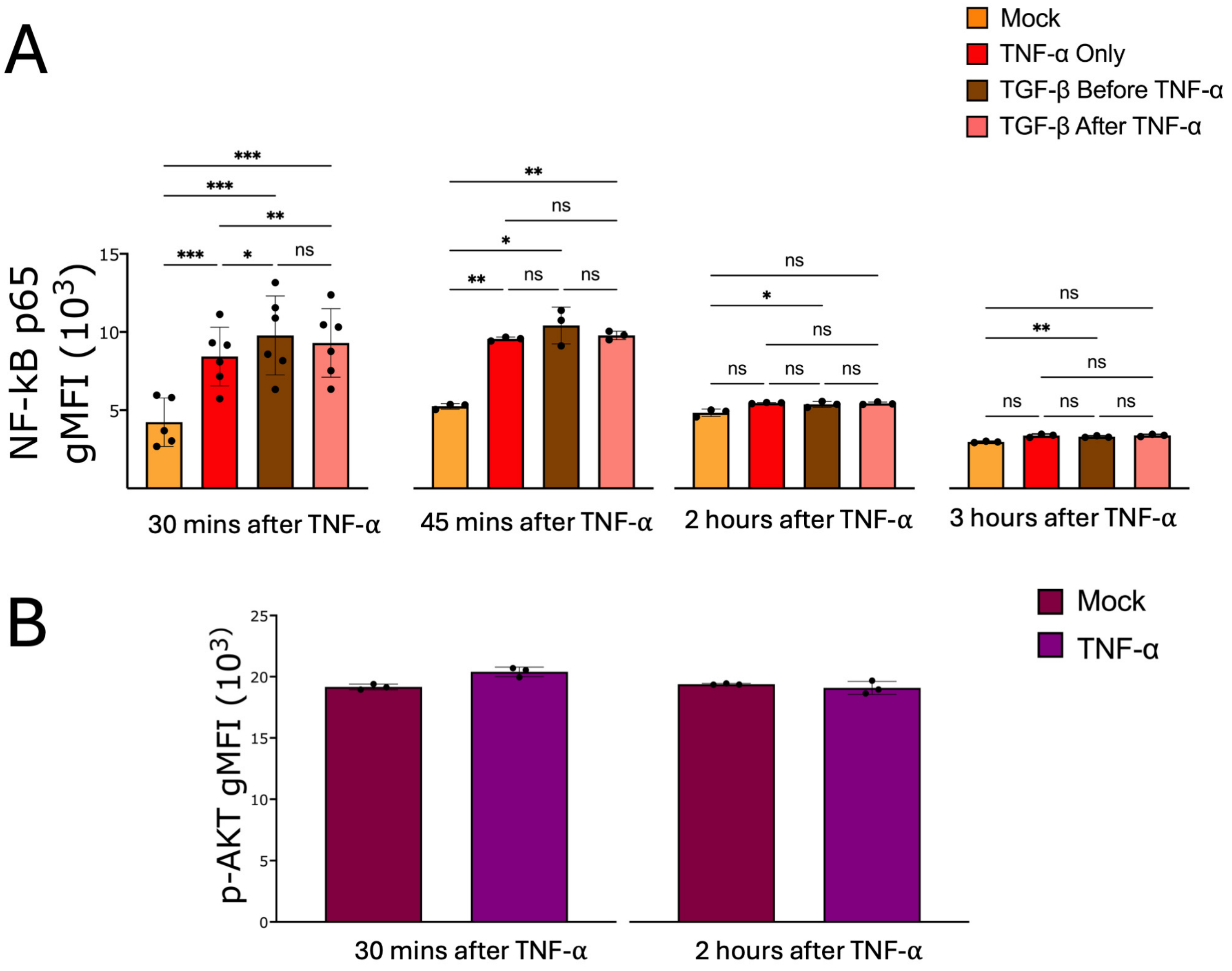

Supplemental Figure S3. TGF-β inhibits latency reversal irrespective of dosage timing and without inhibiting classical reactivation pathways

A) Flow cytometry data for NF-kB p65 where OM10.1 cells were stimulated with TNF-α and treated with TGF-β either 1 hour before or 1 hour after reactivation, with unstimulated cells as a baseline control. Each condition was run in technical triplicate, and readings were taken at 30 minutes, 45 minutes, 2 hours, and 3 hours post-stimulation. Comparisons were made using two-way ANOVA with Tukey's correction for multiple comparisons (*$p < 0.05$, **$p < 0.01$, ***$p < 0.001$)

B) Flow cytometry data for p-AKT from OM10.1 cells ± TNF-α stimulation. Each condition was run in technical triplicate, and readings were taken at 30 minutes and 2 hours post-stimulation.

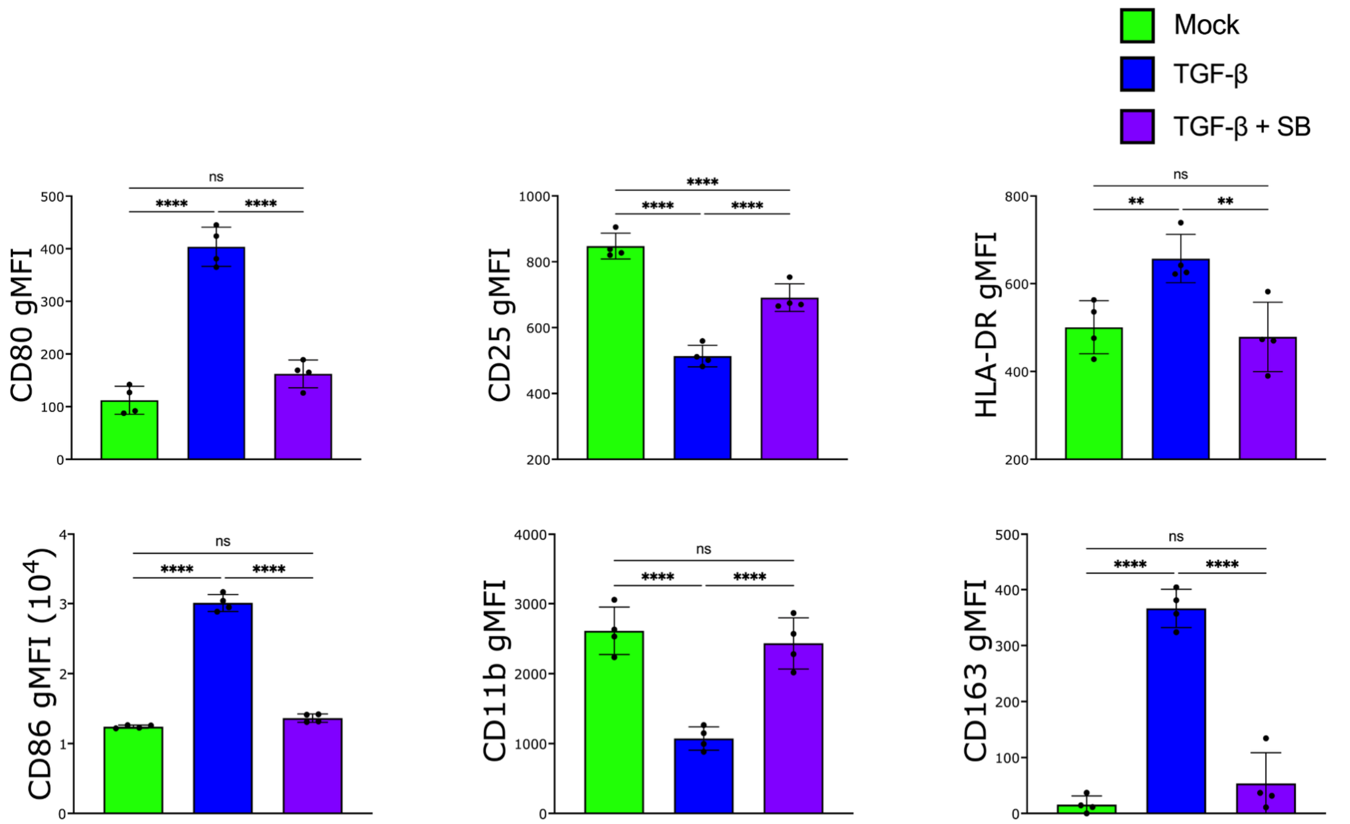

Supplemental Figure S4. TGF-β alters markers of myeloid maturation and APC function

OM10.1 cells were stimulated with TNF-α and then treated with TGF-β or TGF-β + SB 525334 before being stained for common markers of myeloid activation and function as read by flow cytometry. Each condition was run in technical triplicate, and the data was compared using two-way ANOVA with Tukey's correction for multiple comparisons (**$p < 0.01$, ****$p < 0.0001$)

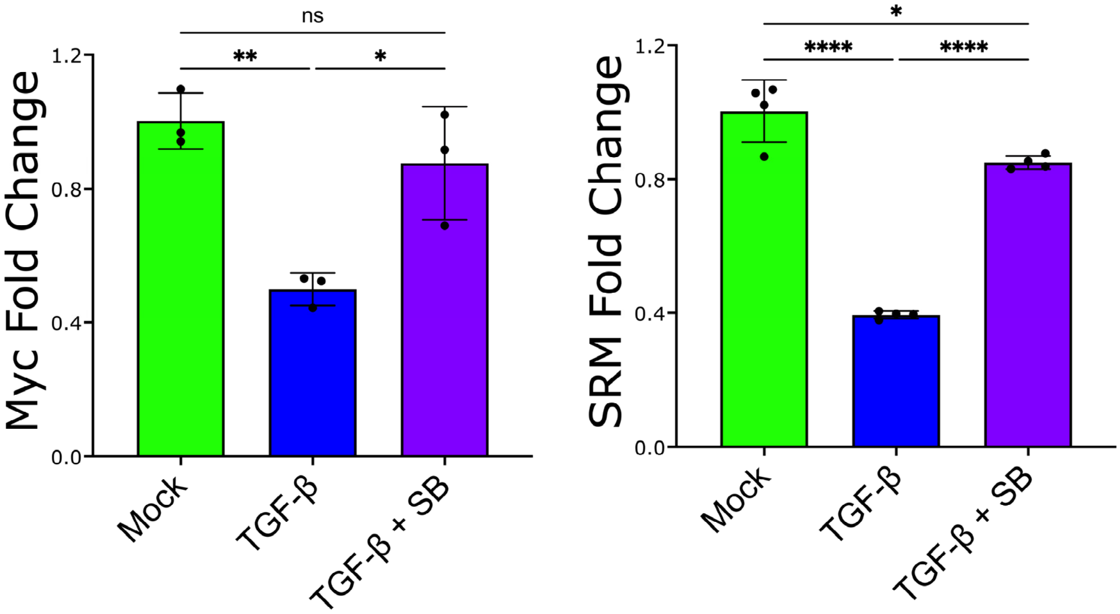

Supplemental Figure S5. TGF-β decreases transcripts linked to the *c-Myc* target pathway

qPCR data generated from cDNA synthesized from RNA isolated from OM10.1 cells stimulated with TNF-α and treated with either TGF-β or TGF-β + SB525334, with each condition run in either technical triplicates (Myc) or quadruplicates (SRM). Transcripts were analyzed using the $\Delta\Delta C_t$ SybrGreen method with the TNF-α only condition being used as the fold change baseline. GAPDH was used as the housekeeping gene for $\Delta\Delta C_t$ analysis. Comparisons were made using two-way ANOVA with Tukey's correction for multiple comparisons (*$p < 0.05$, **$p < 0.01$, ****$p < 0.0001$)

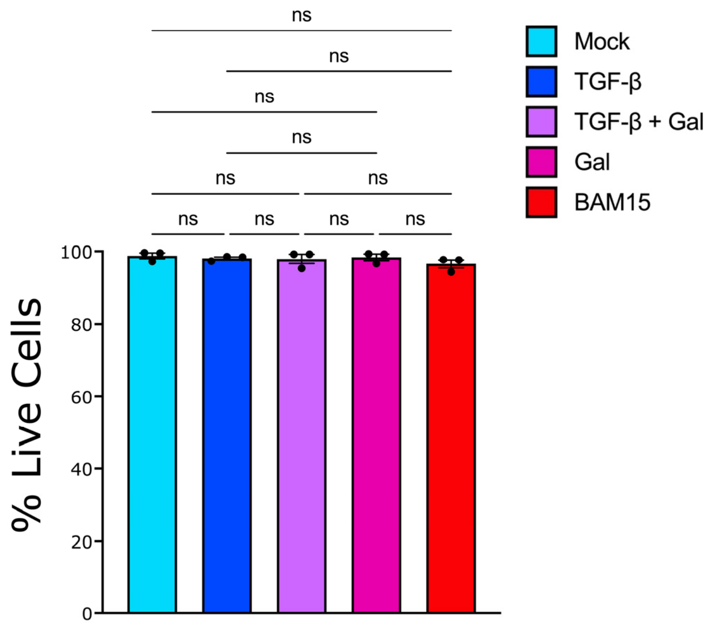

Supplemental Figure S6. Reactivation treatment conditions do not alter cell viability

FVS440UV viability dye flow cytometry data from TNF-α-stimulated OM10.1 cells treated with TGF-β, Gal, TGF-β + Gal, or the mitochondrial uncoupler BAM15 (n = 3 technical replicates per condition). All comparisons were made by two-way ANOVA with Tukey's correction

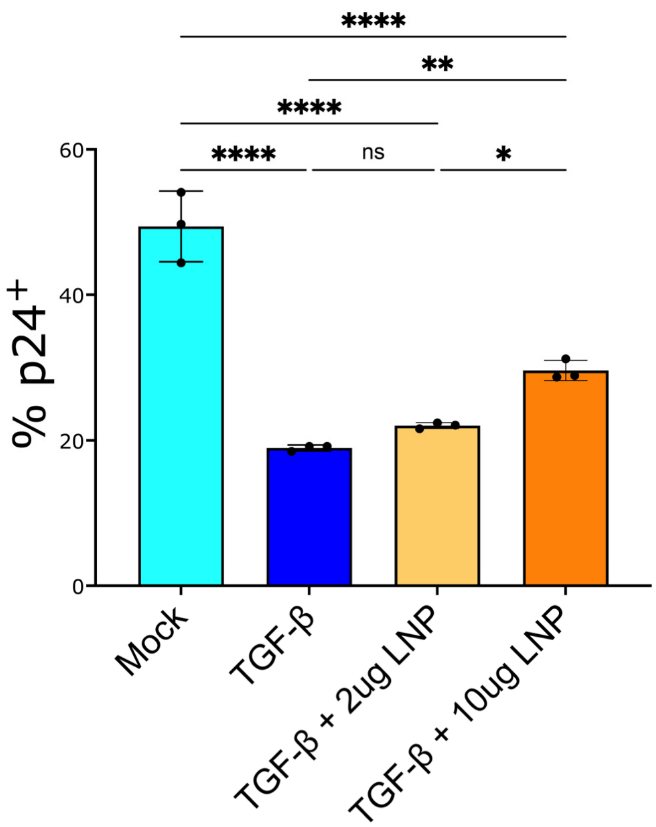

Supplemental Figure S7. LNP transfection of *c-Myc*

p24 flow cytometry data from cells transfected via LNP coated *c-Myc* mRNA, using two different LNP concentrations. The cells were stimulated with TNF-α and TGF-β, with non-transfected cells stimulated with TNF-α ± TGF-β as a control. Each condition was run in technical triplicate. Statistical comparisons were made using two-way ANOVA with Tukey's correction for multiple comparisons (*p < 0.05, **p < 0.01, ****p < 0.0001)
